# Rotavirus Increases Levels of Lipidated LC3 Supporting Accumulation of Infectious Progeny Virus without Inducing Autophagosome Formation

**DOI:** 10.1371/journal.pone.0095197

**Published:** 2014-04-15

**Authors:** Francesca Arnoldi, Giuditta De Lorenzo, Miguel Mano, Elisabeth M. Schraner, Peter Wild, Catherine Eichwald, Oscar R. Burrone

**Affiliations:** 1 Department of Medicine, Surgery and Health Sciences, University of Trieste, Trieste, Italy; 2 International Centre for Genetic Engineering and Biotechnology (ICGEB), Padriciano (Trieste), Italy; 3 Institute of Veterinary Anatomy, University of Zürich, Zürich, Switzerland; 4 Institute of Virology, University of Zürich, Zürich, Switzerland; Juntendo University School of Medicine, Japan

## Abstract

Replication of many RNA viruses benefits from subversion of the autophagic pathway through many different mechanisms. Rotavirus, the main etiologic agent of pediatric gastroenteritis worldwide, has been recently described to induce accumulation of autophagosomes as a mean for targeting viral proteins to the sites of viral replication. Here we show that the viral-induced increase of the lipidated form of LC3 does not correlate with an augmented formation of autophagosomes, as detected by immunofluorescence and electron microscopy. The LC3-II accumulation was found to be dependent on active rotavirus replication through the use of antigenically intact inactivated viral particles and of siRNAs targeting viral genes that are essential for viral replication. Silencing expression of LC3 or of Atg7, a protein involved in LC3 lipidation, resulted in a significant impairment of viral titers, indicating that these elements of the autophagic pathway are required at late stages of the viral cycle.

## Introduction

Viruses are known to induce macroautophagy (hereafter referred to as autophagy) in several different ways, which are either dependent on virus interaction with surface receptors or on viral replication. Autophagy is a homeostatic process that maintains equilibrium of cells by degrading damaged organelles and long-lived proteins and recycling cellular components [1]. Beyond this homeostatic function, in stress conditions autophagy represents an adaptation mechanism promoting cell survival [2]. Autophagy-mediated degradation is achieved through formation of double or multi-membrane structures called autophagosomes, which fuse with lysosomes creating auto(phago)lysosomes, in which degradation takes place. The delivery of cellular material to autophagosomes is both non-specific (“bulk autophagy”) and selective (“selective autophagy”). This latter depends on the activity of several adaptors (e.g. p62, NBR1, NDP52, ALFY, Nix) that deliver specific cargos to autophagosomes [3]. Many aspects of the molecular mechanisms of autophagy, from autophagosome formation to maturation and fusion with lysosomes, still remain obscure. In most cellular settings the autophagic stimulus inhibits the mTOR complex, which is a negative regulator of autophagy through inactivation of the ULK1/2 kinase complex. When the mTOR complex is inhibited, the ULK1/2 kinase complex recruits autophagy-related proteins (Atg) to the site of nucleation of the autophagosome precursor (phagophore) [4]. The same complex also regulates the fusion of autophagosomes with lysosomes [5]. Vesicle expansion and completion are mediated by two ubiquitin-like conjugation systems: one involves the covalent conjugation of Atg12 to Atg5, with the help of the E1-like enzyme Atg7 and the E2-like enzyme Atg10; the second involves conjugation of phosphatidylethanolamine to one of the five members of the microtubule-associated protein 1 light chain 3 (LC3) gene family, LC3B (hereafter referred to as LC3) [6]. LC3 is initially produced as a precursor that is processed through the sequential action of the protease Atg4, which cleaves the C-terminus generating LC3-I, and of Atg7 and Atg3, which generate the lipidated form LC3-II. This latter is the autophagic vesicle-associated form and is generally used as a marker of autophagosomes [7]. Beyond being a marker, LC3-II is involved in the expansion and closure of autophagosomes and also in the delivery of cargo in the selective autophagy [5]. Once in the autolysosome, LC3-II is partly degraded by lysosomal proteases [8] and partly delipidated and recycled [9].

During viral infections, autophagy may have either an antiviral or a proviral role, with a large variety of mechanisms described. Several viruses have evolved mechanisms either to escape or to co-opt elements of the autophagic pathway for their own benefit (for a review see refs. [10], [11]). The knowledge of virulence factors that interfere with autophagy may help to gain insights into the regulation of autophagy and into its manipulation for therapeutic purposes. In this regard, a recent study described the therapeutic potential against several human pathogens (HIV-1, West-Nile virus, chikungunya virus, and the bacterium *L. monocytogenes*) of a peptide derived from the region of the cellular autophagy inducer Beclin-1 targeted by the HIV-1 anti-autophagic maturation protein Nef. Investigation of cellular interacting partners of that peptide led to the identification of a new negative regulator of autophagosome formation called GAPR-1 [12].

In this report, we investigated the induction of autophagy and its role during infection with rotavirus (RV), the main etiologic agent of gastroenteritis in infants and children worldwide. Despite the introduction of two attenuated oral vaccines in 2006, gastroenteritis caused by RV is still responsible for about 453,000 infant deaths annually in developing countries [13], where the vaccine efficacy is extremely low [14]. RV belongs to the family *Reoviridae*, which includes non-enveloped viruses with a segmented genome of double-stranded RNA (dsRNA), and with an exclusively cytoplasmic replication cycle. During entry into the host cell, the virion (a Triple-Layered Particle, TLP) loses the outermost of its three concentric protein layers and becomes a Double-Layered Particle (DLP), which is transcriptionally active. Viral transcripts act both as messengers for the synthesis of viral proteins and as templates for the synthesis of new dsRNA genome segments. Genome replication and assembly of progeny DLPs occur in cytoplasmic inclusion bodies called viroplasms, from which DLPs bud into the endoplasmic reticulum, where viral particle maturation occurs leading to mature TLPs [15]. The viral non-structural protein NSP5 is essential for viroplasm formation and thus for viral replication [16]–[18]. Budding of newly assembled DLPs into the ER is instead mediated by the viral non-structural protein NSP4, which is inserted into the ER membrane and acts as a receptor for DLPs [19]. Virus release has been described to occur either by cell lysis or by exocytosis [20], [21].

Recently, it has been reported that NSP4 induces autophagy through its well-known capacity of releasing calcium from the ER, which stimulates a CaMKK-β/AMPK (calcium/calmodulin-dependent kinase kinase-β/5′-adenosine monophosphate-activated protein kinase) dependent signaling pathway responsible for induction of autophagy [22], [23]. Here we show that accumulation of lipidated LC3 induced by actively replicating RV does not correlate with increased autophagosome formation while exerting a pro-viral role favouring accumulation of infectious progeny virus.

## Results

### Rotavirus induces accumulation of lipidated LC3 but not of autophagosomes

**Figure 1:**
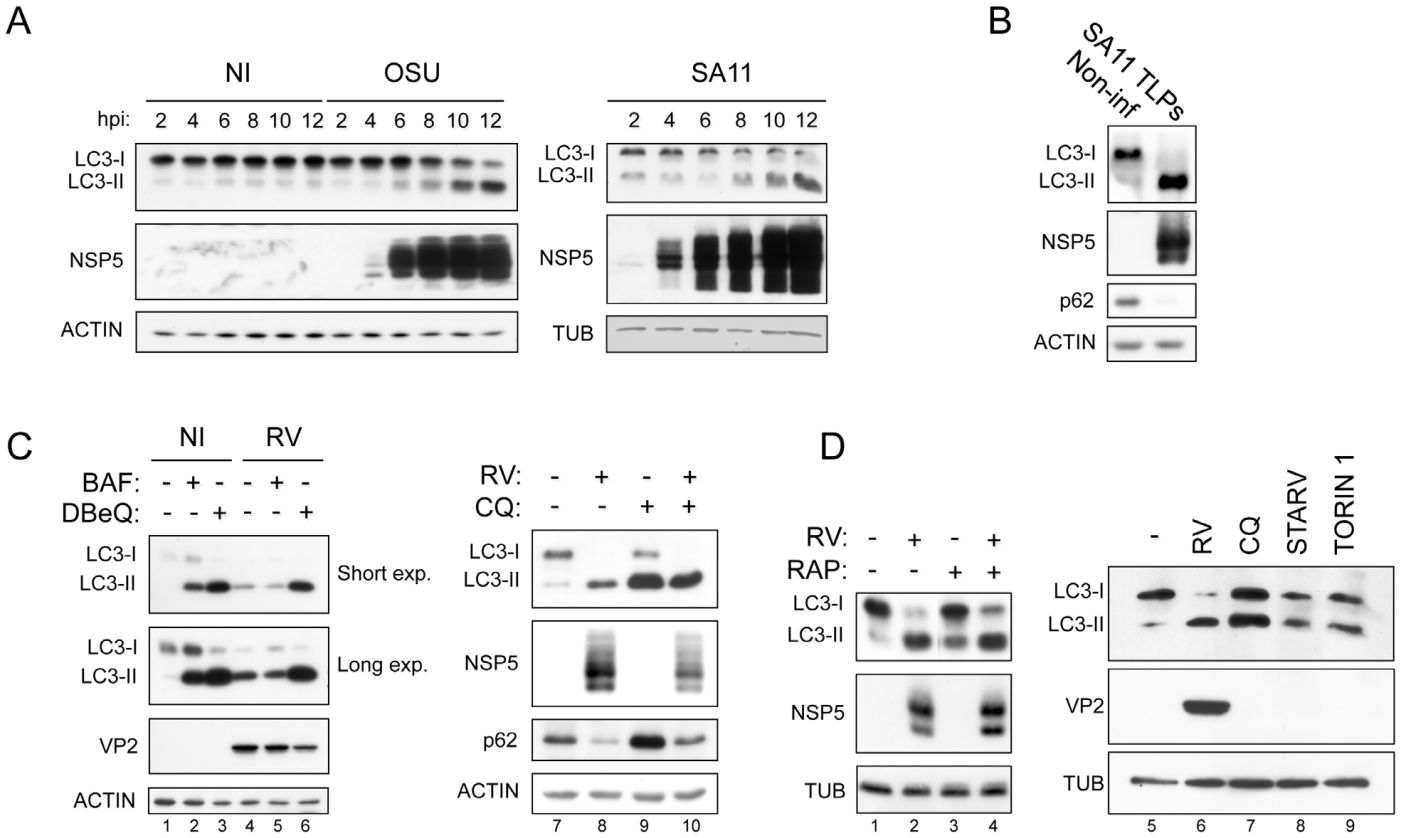
LC3 lipidation induced by RV infection. A) Western blot of extracts from non-infected (NI) and OSU- or SA11-infected MA104 cells at different times post-infection. Crude viral preparations were used for infection. B) As in A, using SA11 purified triple-layered particles (TLPs) (13 hpi). C–D) Western blots of extracts from NI and OSU-infected cells at 13 hpi, treated or not with BAF (0.1 µM), DBeQ (15 µM), CQ (50 µM), RAP (0.1 µM) or torin 1 (0.25 µM), or maintained under starvation conditions, as indicated.

Infection of MA104 cells with RV resulted in an increase of lipidated LC3 but not in autophagosome accumulation, as revealed by different criteria: i) quantitation of LC3-II levels by Western blot (WB), ii) visualization of LC3 by confocal microscopy and iii) visualization of autophagosomes by electron microscopy (EM). As shown in Figure 1, for two different RV strains, porcine OSU and simian SA11, LC3-II levels were highly increased upon infection initiating from 6 to 8 hours post-infection (hpi) and reaching maximum values at a late time post-infection (12 hpi). This effect was observed with both crude viral preparations (Fig. 1A) and CsCl gradient purified TLPs (Fig. 1B). The concomitant diminished level of LC3-I is indicative of an increased conversion of LC3-I into LC3-II. However, a dramatic increase in LC3-II levels may also reflect inhibition of LC3-II degradation. To investigate this aspect further, three autophagy inhibitors acting at post-sequestration steps, chloroquine (CQ), bafilomycin A1 (BAF) and *N2,N4*-dibenzylquinazoline-2,4-diamine (DBeQ), were added at 1 hpi (or at 9 hpi in experiments not shown) and the level of LC3-II monitored at 13 hpi by WB. As expected, in non-infected cells all three inhibitors increased LC3-II levels (Fig. 1C, lanes 2, 3, and 9); in RV-infected cells a further increase was apparent with DBeQ and CQ, but not with BAF (Fig. 1C, lanes 5 and 6 compared to lane 4 and lane 10 compared to lane 8), indicating that a substantial amount of LC3-II is still degraded during RV infection.

**Figure 2:**
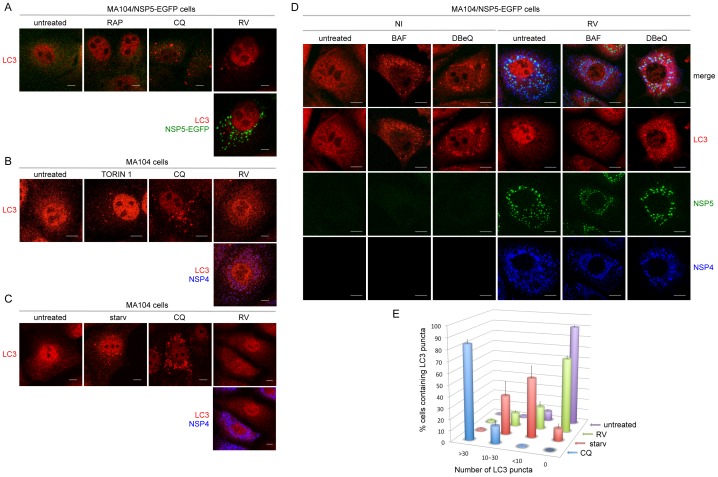
Autophagosomes in RV-infected cells. A–D) Confocal immunofluorescence of non-infected (NI) and RV-infected (OSU strain, 13 hpi; MOI: 0,5 in A–C; 5 in D) MA104/NSP5-EGFP cells (A, D) and MA104 cells (B, C). Cells were treated or not with BAF (0.1 µM), DBeQ (15 µM), CQ (50 µM), RAP (0.1 µM), or torin 1 (0.25 µM), or maintained under starvation conditions (starv), as indicated. Autophagosomes were visualized with an anti-LC3 antibody (red). In A and D viroplasms were visualized using NSP5-EGFP (green). In B and C NSP4 was visualized using an anti-NSP4 antibody (blue). Single optical sections are shown. Scale bar is 5 µm. Images are representative of three independent experiments in which at least 150 cells per each experimental condition were analyzed. E) Quantification of LC3 puncta: the results are expressed as mean ±SEM from at least three independent samples for each experimental condition.

Activation of autophagy was further investigated by confocal microscopy with an anti-LC3 antibody that recognizes both LC3-I and LC3-II. Viroplasms were visualized either using the MA104 cell line stably expressing NSP5-EGFP (MA104/NSP5-EGFP), which upon RV infection re-localizes to viroplasms [24], or with anti-NSP5 antibodies in wild-type MA104 cells. A very low number of LC3 puncta was observed in virus-infected cells (Fig. 2), which was similar to that of untreated cells and also to cells treated with the autophagy inducer rapamycin (RAP) (Fig. 2A). This drug induced only a modest increase of LC3-II levels in MA104 cells, in both non-infected and infected cells (Fig. 1D, lanes 3 and 4). Stronger autophagy induction was obtained either through starvation of both serum and amino acids or through treatment with torin 1, a potent and selective inhibitor of mTOR (Fig. 1D). As expected, both treatments induced the formation of a number of LC3 puncta that was higher than that observed in untreated cells and much lower than that in CQ-treated cells (Fig. 2B–C). Importantly, the number of LC3 puncta in RV-infected cells was closer to that of untreated cells (Fig. 2B–C) and clearly much lower than that in cells treated with CQ (Fig. 2A–C), BAF or DBeQ (Fig. 2D). Quantification of LC3 puncta was carried out in three independent experiments and confirmed that RV infection does not significantly increase the number of autophagosomes in MA104 cells (Fig. 2E).

**Figure 3:**
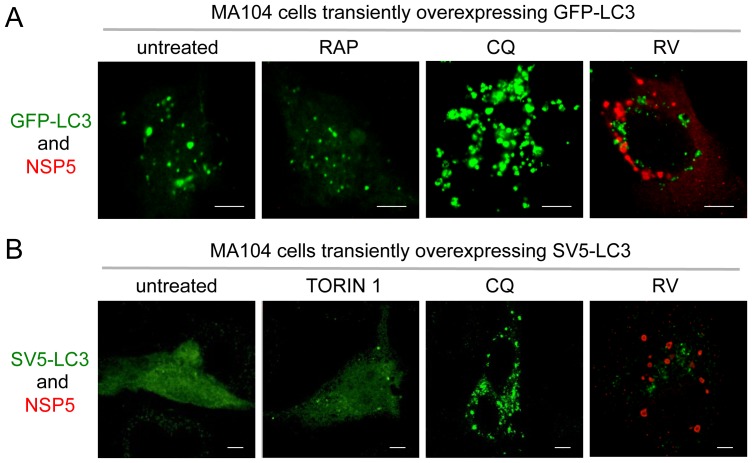
GFP-LC3 and SV5-LC3 fusion constructs in RV-infected cells. Confocal immunofluorescence of MA104 cells transiently over-expressing the fusion construct GFP-LC3 (A) or SV5-LC3 (B). Cells were untreated, RV-infected (OSU strain; MOI, 5; 13 hpi) or treated from 1 hpi to 13 hpi with RAP (0.1 µM), CQ (50 µM), or torin 1 (0.25 µM), as indicated. Viroplasms were visualized with an anti-NSP5 antibody (red) and the fusion constructs with the GFP fluorescence in A and with an anti-SV5 antibody in B (green). Single optical sections are shown. Scale bar, 10 µm. Images are representative of three independent experiments in which at least 150 cells per each experimental condition were analyzed.

Autophagosome formation was also analyzed by transient over-expression of two different versions of N-terminally tagged LC3, GFP-LC3 [7] and SV5-LC3. This latter construct was validated through Western blot analysis and co-localization with endogenous LC3 in immunofluorescence of CQ-treated MA104 cells (Fig. S1). In RV-infected cells the number and size of LC3 puncta resembled that of untreated or RAP- or torin 1-treated cells and not of CQ-treated cells (Fig. 3). Altogether, these data indicate that RV behaves neither as an inducer of autophagosome formation nor as a CQ-like inhibitor of autophagy and that, surprisingly, the increased levels of LC3-II do not correlate with an increased number of autophagosomes.

**Figure 4:**
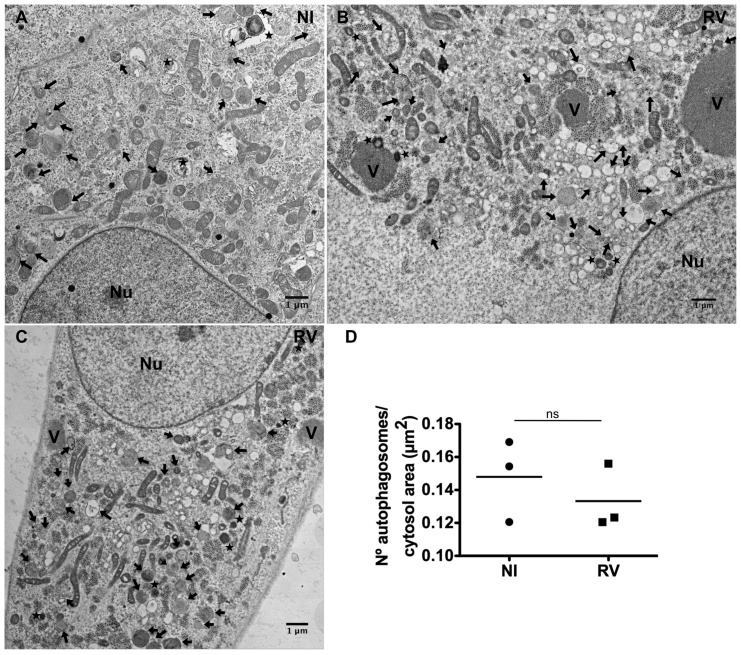
Electron microscopy of autophagosomes in RV-infected cells. High-definition electron microscopy of non-infected (A) and RV-infected (SA11 strain; MOI, 250 VFU/ml) (B and C) MA104 cells at 14 hpi. V, viroplasms; Nu, nucleus; black arrows, AVi (early/initial autophagic vacuoles corresponding to autophagosomes); stars, AVd (late/degradative autophagic vacuoles including amphisomes and autolysosomes). Scale bars are 1 µm. D) Quantification of autophagosomes in non-infected (NI) and RV-infected MA104 cells (14 hpi). The data correspond to the mean of three-independent experiments with 25 cells per experimental point. Student's t-test, ns, p>0.05.

To further investigate whether RV infection modifies the number of autophagosomes or autolysosomes, we carried out EM studies of RV-infected cells at late times post-infection (14 hpi). Neither an increase in terminal autolysosomes nor an accumulation of double-membrane vacuoles was observed (Fig. 4), thus confirming that the increased LC3-II levels in RV-infected cells do not lead to an increased number of autophagosomes.

**Figure 5:**
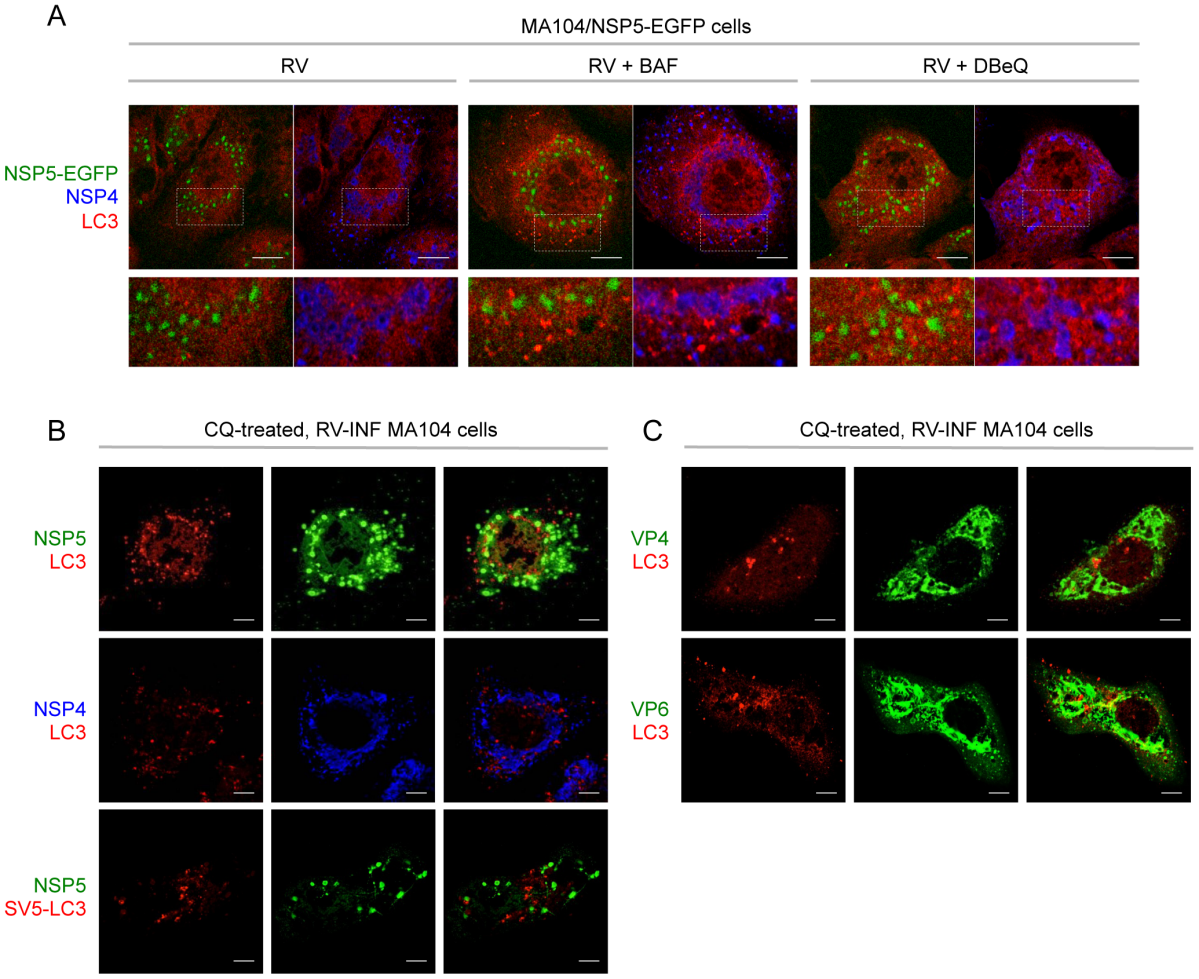
Cellular localization of LC3 in RV-infected cells. Confocal immunofluorescence of RV-infected (OSU strain, 13 hpi; MOI, 5) MA104/NSP5-EGFP cells (A) and MA104 cells (B–C). In B, bottom row, cells were transiently transfected with the pSV5-LC3 construct. Where indicated, autophagy inhibitors (BAF, DBeQ, CQ) were used. Autophagosomes were visualized with an anti-LC3 antibody (red) in A, in the upper and middle rows of B and in C, and with an anti-SV5 antibody (red) in the bottom row of B. Viroplasms were visualized using the fluorescence of NSP5-EGFP in A and with an anti-NSP5 antibody (green) in B. NSP4, VP4 and VP6 were visualized using anti-NSP4 (blue), anti-VP6 (green) and anti-VP4 (green) antibodies, respectively. In A, bottom row shows magnification of insets indicated by dotted squares. Single optical sections are shown. Scale bar, 5 µm. Images are representative of three independent experiments in which at least 150 cells per each experimental condition were analyzed.

Notably, from the images in Figure 2 it was already apparent that LC3 puncta in virus-infected cells were not co-localizing with NSP4 and viroplasms. This is in sharp contrast with the data previously published by Berkova et al. (2006) and Crawford et al. (2012). We thus performed immunofluorescence studies in both MA104/NSP5-EGFP and wild-type MA104 cells as shown in Figure 5. Viroplasms were visualized through NSP5-EGFP (Fig. 5A) or with an anti-NSP5 antibody (Fig. 5B), while LC3 was detected with a specific antibody (Fig. 5A and 5B, upper and middle panels) or following over-expression of SV5-LC3 (Fig. 5B, bottom panel). The results in both types of cells confirmed lack of co-localization of LC3 with NSP4 and viroplasms. In addition, we found that LC3 does not co-localize with two structural viral proteins: VP4, a protein only present in the outer layer of mature particles (TLPs), and the middle layer protein VP6, which is a marker of DLPs (particularly at late time points post-infection) as it is not accessible to antibodies in mature particles (Fig. 5C). Therefore, our data suggest that LC3-II interacts directly neither with TLPs (VP4) nor with DLPs (VP6).

Collectively, our data demonstrate that RV strongly induces accumulation of lipidated LC3, which nevertheless does not lead to increased autophagy or to accumulation of autophagosomes. The further increase of LC3-II upon CQ or DBeQ treatment together with a number of autolysosomes comparable to that of non-infected cells suggest that partial degradation of LC3-II, and therefore autophagy, still takes place during RV replication.

### Active rotavirus replication is required for accumulation of lipidated LC3

**Figure 6:**
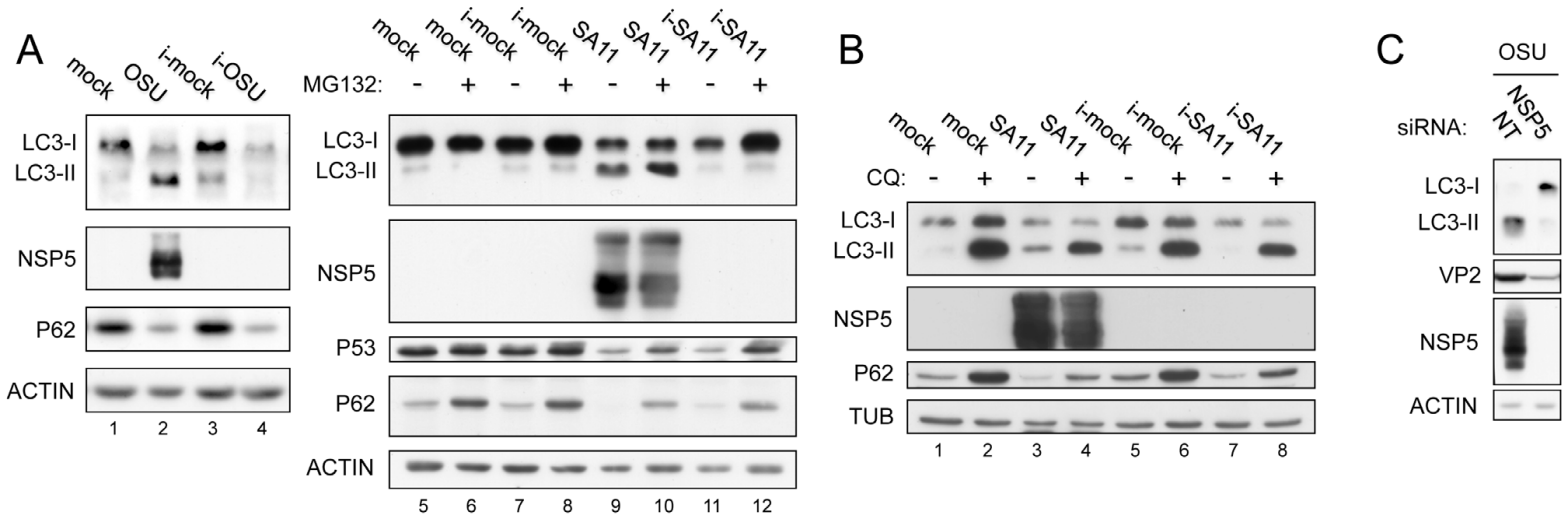
Requirement of virus replication for accumulation of lipidated LC3. A–B) Western blots of extracts from MA104 cells infected or not with antigenically intact inactivated RV particles (i-OSU or i-SA11, 13 hpi) and untreated or treated with MG132 (5 µM, added at 1 h before infection) in A and with CQ (50 µM, added at 1 h after infection) in B. Since virus inactivation was performed on crude preparations, equal amounts of lysates derived from non-infected cells were psoralen-treated and UV-exposed and used as mock-infection controls (i-mock). C) Western blot of extracts from MA104 cells transfected with the indicated siRNAs and infected with RV (OSU strain; MOI, 5; 13 hpi) at 48 h after transfection.

In order to establish whether the increase of lipidated LC3 requires viral replication, we performed experiments with inactivated viral particles and with a siRNA specific for the non-structural protein NSP5, which is essential for virus replication. In both assays, LC3-II levels were determined by WB. As shown in Figure 6A, antigenically intact inactivated RV particles (i-OSU and i-SA11) did not increase LC3-II levels. This may reflect either the lack of LC3 lipidation or an increased LC3-II degradation following lipidation. The fact that treatment with CQ did not show an increase of LC3-II in inactivated SA11-infected cells as compared to the i-mock control (Fig. 6B, compare lanes 6 and 8) indicates that the autophagic flux (increased LC3-II degradation) is not enhanced upon RV binding (and possibly entry) to the host cell. On the other hand, treatment with CQ led to comparable levels of LC3-II in SA11- and i-SA11-infected cells (Fig. 6B, compare lanes 4 and 8), indicating that LC3-II rescue is lower when the virus is actively replicating. This suggests that RV replication subtracts part of LC3-II to degradation. Since a reduction of p62 levels is frequently used as an additional criterion to establish completion of the autophagic flux, we determined its level in cells infected with both actively replicating and inactivated RV. Surprisingly, while replicating RV induces both increase of LC3-II and decrease of p62 (Fig. 6A, lanes 1,2, and 5,9), inactivated-RV (i-OSU and i-SA11) did cause only significant reduction of p62 (Fig. 6A, lanes 3,4 and 7,11). However, in mock-infected or virus-infected MA104 cells both CQ and the proteasome inhibitor MG132 partially rescued p62, while MG132 essentially did not affect LC3-II levels (Fig. 6A, B). Thus, because of the extensive degradation by the proteasome, p62 levels cannot be used in our model to reliably detect autophagic activity.

Interestingly, inactivated RV as well as replicating virus was able to activate proteasomal degradation, as shown by the decrease of p53, a common marker of proteasomal activity, and also by the rescue of p62 and p53 upon MG132 treatment (Fig. 6A).

The results with the inactivated viruses indicate that active replication is needed for accumulation of LC3-II. This was further confirmed by NSP5 knock down experiments, in which concomitantly with compromised viral replication LC3-II was almost undetectable (Fig. 6C).

Altogether, the data shown above indicate that accumulation of LC3 into its lipidated form in RV-infected cells requires active virus replication.

### Lipidated LC3 supports production of infectious RV particles

**Figure 7:**
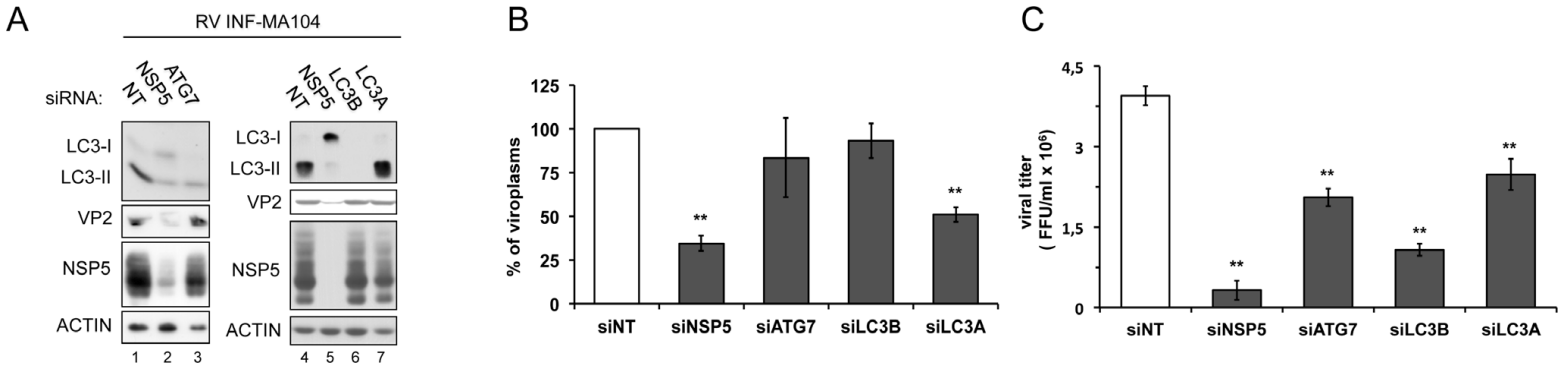
Proviral effect of LC3 lipidation. MA104 cells were transfected with the indicated siRNAs and at 48(OSU strain; MOI, 5) for 13 h (A, B) or 24 h (C). A) Western blot of cellular extracts. B) Quantification of the accumulation of viroplasms per cell. C) Viral titers obtained from each condition. The results are expressed as mean ±SEM from at least three independent samples for each experimental condition, and in B data were normalized as percentage from the control. In all figures: siNT, control non-targeting siRNA. t-test, **, p<0.01.

Next, we investigated the role of LC3 lipidation in the RV replication cycle. To this aim, the effect of silencing either of the two essential autophagy effectors Atg7 and LC3 was studied in RV-infected cells. Atg7 silencing was indirectly assessed through the reduced levels of LC3-II in both RV-infected cells (Fig. 7A) and in non-infected RAP-treated cells (Fig. S2). In these experiments we also included a siRNA specific for LC3A, another member of the LC3 gene family with a not well-defined role in autophagy. Three different parameters were determined: i) accumulation of viral proteins at late times post-infection, ii) number of viroplasms per cell and iii) yield of infectious viral particles. As shown in Figure 7A, the amount of viral proteins VP2 and NSP5 accumulated upon depletion of Atg7, LC3B or LC3A was not significantly altered. Interestingly, LC3A depletion had no effect on LC3B lipidation. The number of viroplasms per cell determined at 13 hpi was found essentially unchanged in the absence of LC3B or Atg7 and reduced upon depletion of LC3A (Fig. 7B). Viroplasms were monitored using the MA104/NSP5-EGFP cell line [24]. The same cellular system was used to measure virus yields in terms of fluorescence forming units (FFU)/ml of viral preparations obtained from cells transfected with siRNAs directed to Atg7, LC3B or LC3A and infected with RV for 24 hours. As shown in Figure 7C, viral titers were significantly lower when RV was grown in cells lacking LC3B or Atg7, and with a less pronounced effect in cells lacking LC3A. In conclusion, the data shown for LC3B and Atg7 clearly indicate that RV benefits from LC3 lipidation with an effect at later stages of the virus cycle favouring accumulation of infectious progeny virus.

## Discussion

The involvement of autophagy in viral infections has been recently uncovered for a variety of RNA and DNA viruses. Depending on the virus and the host cell, autophagy may exert antiviral roles by clearing cells from viral particles or by stimulating the innate immune system or, alternatively, be exploited by viruses to facilitate their own replication through different mechanisms. For instance, many picornaviruses take advantage of autophagosomes as platforms for genome replication [25]–[28]. Instead, coronaviruses use the sole non-lipidated form of LC3 (LC3-I) for the genesis of replication vesicles [29], while HCV initiates and blocks the last steps of autophagy in order to exploit some autophagy genes (such as Atg5, Atg7 and Beclin-1) for efficient replication [30], [31]. Other viruses require autophagy elements for virion assembly (e.g. HIV, HBV) or induce autophagy to modulate cellular survival (human parvovirus B19) or metabolism (Dengue virus) (for a review see ref. [10]).

In the case of RV it has been recently reported that the viral non-structural protein NSP4 initiates autophagy to guarantee correct localization of the viral ER-associated proteins VP7 and NSP4 around viroplasms [23]. Here we report novel findings that in part diverge from the proposed scenario of autophagy during RV infection.

We observed increased levels of lipidated LC3 with two different RV strains (OSU and SA11), which did not result in an increased autophagy (no increase in autophagosomes or autolysosomes). The accumulation of LC3-II and the concomitant reduction of LC3-I started at about 6 hpi and became particularly pronounced at 12 hpi.

The increase of LC3-II was most likely the consequence of a higher conversion of LC3-I into LC3-II. In fact, we observed (i) a concomitant decrease of LC3-I and (ii) a further LC3-II increase when using two different inhibitors of autophagy, namely CQ and DBeQ. Since CQ inhibits autophagy by increasing the lysosomal pH, which leads to inhibition of both fusion of autophagosomes with lysosomes and lysosomal protein degradation, and DBeQ inhibits the ATPase activity of p97 [32], which plays an important role in autophagosome maturation [33], [34], their effects in infected cells suggest that a basal level of the autophagic flux is maintained. Bafilomycin A1, however, which inhibits acidification of lysosomes by acting on the vacuolar type H^+^-ATPase (V-ATPase), did not cause any LC3-II increase in infected cells. One possibility is that virus infection moderately impairs LC3-II degradation to an extent similar to BAF. Indeed, BAF showed a lower increase of LC3-II in non-infected cells as compared to CQ and DBeQ and a lower number of accumulated autophagosomes than that observed in CQ- and DBeQ-treated cells (see Fig. 2D). This infection-dependent activity is also suggested by the lower LC3-II rescue obtained with CQ in cells infected with actively replicating SA11 as compared to inactivated SA11. Therefore, it remains still unclear whether the robust increase in the LC3-II levels observed upon RV infection is the consequence of enhanced LC3-I lipidation or partial inhibition of LC3-II degradation, or a combination of both. What is clearly indicated by our data is that neither autophagosomes nor autolysosomes accumulated in infected cells, as shown by immunofluorescence experiments and by EM studies. Crawford et al. (2012), over-expressing the tandem construct RFP-GFP-LC3 in RV-infected cells (where only the RFP fluorescence persists at the autolysosomal pH), proposed a block of the autophagic flux at the level of fusion between autophagosomes and lysosomes. However, over-expression of LC3 fusion constructs may be troublesome, as we verified by detecting many puncta containing GFP-LC3 in both non-infected and infected cells (Fig. 3A). These puncta most likely represent an artefact due to GFP-LC3 over-expression, as no such situation corresponds to what is observed with endogenous LC3. In this regard, there is evidence indicating that they may in part represent aggregates as a consequence of overexpression [35], [36]. In addition to the troublesome over-expression of LC3 fusion constructs, also the use of p62 as an autophagy marker needs to be evaluated with extreme caution. Specifically, in RV-infected MA104 cells p62 was significantly degraded via proteasome, as demonstrated using the proteasome inhibitor MG132. It is therefore mandatory to use reliable methods to monitor autophagy in order to avoid misinterpretations. Here, we used an N-terminal SV5 tag [37] as an alternative to the GFP fusion. Possibly because of the small size of the SV5 tag (11 aa), over-expression of SV5-LC3 did not cause formation of artefactual puncta in untreated cells. This construct provided further evidence of both lack of autophagosome accumulation in RV-infected cells and association of LC3-II to viroplasms.

We showed that accumulation of LC3-II in RV-infected cells was totally dependent on viral replication. In fact, infecting cells with inactivated viral particles or silencing NSP5, a viral protein that is essentially required for viral replication [17], did not increase LC3-II levels. In addition, we found that lipidated LC3 plays a positive role exclusively at late stages of the viral cycle. More in detail, LC3 and Atg7 are required for optimal viral yields while their role at early stages (accumulation of viral proteins and viroplasms) seems to be marginal. These data are consistent with the reported observation that inhibition of LC3 lipidation (upon treatment with STO-609, a CaMKK-β inhibitor) impairs recruitment around viroplasms of two viral proteins, NSP4 and VP7, both involved in the final stages of the viral replication cycle [23]. Interestingly, silencing of LC3A partially impaired viroplasm formation without affecting LC3B lipidation, but did not have a strong effect on viral yields, suggesting that LC3A does not have an overlapping function with LC3B. In contrast to other RNA viruses such as coronaviruses and equine arteritis virus that require non-lipidated LC3 to anchor replication complexes to ER membranes [29], [38], RV, which does not need ER-derived membranes for genome replication, requires Atg7 indicating a role for the lipidated form of LC3 in virus morphogenesis. Interestingly, an autophagy-independent role for lipidated LC3 has been recently described in microtubule dynamics [39]. It is therefore possible that the microtubule network might be involved in an LC3-dependent localization of viral proteins participating in the final stages of virus morphogenesis (such as NSP4 and VP7), also considering that such network has been already shown to participate in the dynamics of RV viroplasms [40].

Collectively our data suggest a scenario that somehow differs from what has been previously proposed, in which RV replication: i) induces accumulation of LC3-II; ii) does not lead to accumulation of autophagosomes in spite of large accumulation of lipidated LC3; iii) takes advantage of LC3-II for improved production of infectious progeny virus.

Understanding where LC3-II is targeted, as it is not loaded into autophagosomes, deserves to be explored. In contrast to what Berkova et al. (2006) reported [22], we did not find co-localization of LC3 with NSP4 at all, nor any association of LC3 with viroplasms, regardless of whether LC3 was over-expressed or not. In addition, we did not find co-localization of LC3 with two structural proteins, VP4 and VP6, which can be considered as markers of TLPs and DLPs, respectively. These data suggest that there is no direct interaction of LC3-II with TLPs or DLPs. Notably, the immunofluorescence with anti-VP6 is consistent with the lack of co-localization of LC3 with viroplasms. In fact VP6, like NSP5, is a viroplasm resident protein at early times post-infection while at late times post-infection it is present also outside viroplasms in newly made DLPs, including those already translocated to the ER lumen.

In conclusion, accumulation of lipidated LC3 represents a cellular outcome of viral replication, which does not seem to imply a direct interaction of LC3 with viral proteins or viral particles. Investigating cellular proteins interacting with LC3, and in particular with its lipidated form, in RV-infected cells could help in identifying the targeting site of LC3-II during infection. This would also contribute to elucidate the still poorly understood mechanisms that regulate participation of LC3 in autophagy.

We have recently shown that the ubiquitin-proteasome system (UPS), the second major intracellular protein degradation system, is essential at the early stages of RV replication, in particular for the correct assembly of viroplasms [41]. Interestingly, in this study we report activation of the UPS upon RV infection, which, differently from LC3 lipidation, was independent of viral replication, as it was also induced by inactivated viral particles.

Like many other viruses, RV developed mechanisms to exploit components of the cell host degradative systems to favor its replication at different stages of the viral cycle. Understanding the molecular mechanisms of these virus-host interactions represents an intriguing challenge and may suggest cellular targets for novel antiviral therapies.

## Materials and Methods

### Cells and Viruses

MA104 cells (embryonic African green monkey kidney cells, purchased from the original deposit CRL-2378 of the American Type Culture Collection) were grown in Dulbecco's Modified Eagle's Medium (DMEM) (Life Technologies) containing 10% Fetal Bovine Serum (FBS) (Life Technologies), and 50 µg/ml gentamycin (Biochrom AG).

NSP5-EGFP/MA104 cell line was obtained as described previously [24], [42] and cultured in DMEM containing 10% FBS and 800 µg/ml geneticin (Life Technologies). The simian SA11 (G3, P6[1]) and porcine OSU (G5, P9[7]) strains of rotavirus (RV) were propagated in MA104 cells as described previously [43], [44]. Virus was titered by immunofluorescence of infected NSP5-EGFP/MA104 cells and titers were expressed as “fluorescence forming units” (FFU/ml), as already described [41]. For virus purification, crude extracts of infected MA104 cells were ultracentrifuged, the pellets extracted with Freon (trichlorotrifluoroethane; Sigma) and banded by equilibrium ultracentrifugation in CsCl gradient, essentially as described by Patton et al., 2000 [45].

Inactivation of viral particles was obtained by treatment of viral crude extracts with 40 µg/ml psoralen (Sigma) and long-wave UV exposure for 15 minutes, as previously described [46].

### Plasmids and Chemicals

Cells were treated with the autophagy inhibitors chloroquine (50 µM, Sigma), bafilomycin A1 (0.1 µM, Sigma), *N2,N4*-dibenzylquinazoline-2,4-diamine [32] (15 µM, Sigma), and with the autophagy inducers rapamycin (0.1 µM, LC Laboratories) and torin 1 (0.25 µM, Merck Millipore). Drugs were added at 1 hpi or at 9 hpi in DMEM containing 10% FBS. The proteasome inhibitor MG132 (5 µM, Calbiochem) was added 1 h before infection.

The plasmid encoding the fusion protein GFP-LC3 was kindly provided by Dr. R. Bernasconi (IRB, Bellinzona, Switzerland). pSV5-LC3 was obtained by substituting the GFP coding sequence for a synthetic oligonucleotide encoding the SV5 tag (using the restriction enzymes NheI and BspEI).

### Infections and Transient Transfections

Infection experiments were carried out at an MOI of 5 FFU/cell. For experiments of combined plasmid transfection and RV infection, about 7.5×10^5^ MA104 cells were electroporated with 2 µg DNA plasmid using the Amaxa/Lonza Nucleofector Technology (K-029 program; T solution); at 48 h post-transfection cells were OSU-infected, at 1 hpi provided with serum and at 13 hpi harvested.

For serum and amino acid starvation, cells were deprived of serum overnight and then incubated for 4 h in PBS containing calcium 100 mg/l, magnesium 100 mg/l, and glucose 1 g/l.

For siRNA experiments, 5×10^4^ MA104 cells/well were seeded into 12-multiwell plates and the next day were transfected with 0.1 nmol of annealed duplex siRNA (Sigma) using 5 µl of RNAiMAX Lipofectamine 2000 (Life Technologies) following the manufacturer's instructions. The following siRNAs were transfected: siLC3A 5′-CGGUGAUCAUCGAGCGCUA-3′, siLC3B 5′-CGAACAAAGAGUAGAAGAU-3′, siATG7 5′-GGUCAAAGGAUGAAGAUAA-3′. Control siRNAs were si/SA11 (here referred to as siNT) and si/OSU (here referred to as siNSP5) as described by Campagna et al., 2005 [17]. At 48 h post-transfection cells were infected at the same MOI and collected at either 13 hpi (for viroplasm counting by immunofluorescence or for Western blot analyses) or 24 hpi (for viral titers determination).

Cellular extracts (about 3×10^5^ cells) were prepared with 50 µl of reducing SDS buffer (125 mM Tris-HCl pH 6.8, 6% SDS, 40% glycerol, 5% β-mercaptoethanol, 0.04% bromo phenol blue) and subsequently sonicated with a VialTweeter (Hielscher Ultrasonics GmbH) for 1 min (10 W, pulse 0.5 sec) to disrupt DNA. Typically, 15 µl of supernatant was loaded into SDS-PAGE for Western blot analyses.

### Page and Western Blot Analysis

Cellular extracts were resolved by 14% SDS-PAGE and proteins were transferred to polyvinylidene difluoride membranes (Millipore, IPVH00010). The membranes were incubated with the following antibodies: anti-NSP5 guinea pig serum (1∶10,000), anti-VP2 guinea pig serum (1∶5,000), anti-LC3B mouse monoclonal antibody (1∶2,000, NanoTools), anti-p62 guinea pig serum (1∶1,000, ProgenBiotechnik), anti-p53 mouse monoclonal antibody (1∶5,000, Santa Cruz Biotechnology), anti-SV5 mouse monoclonal antibody, 1∶10,000, anti-α-tubulin mouse monoclonal antibody (1∶3,000, Calbiochem), anti-actin rabbit polyclonal antibody (1∶1,000, Sigma), and HRP-conjugated goat anti-guinea pig (1∶10,000, Jackson ImmunoResearch), goat anti-mouse (1∶5,000, Jackson ImmunoResearch), goat anti-rabbit (1∶5,000, Thermo Scientific Pierce) secondary antibodies. Sera were produced by immunization of guinea pigs as described previously [47]–[49]. Signals were detected by using the enhanced chemiluminescence system (Pierce ECL Western Blotting Substrate, Thermo Scientific).

### Immunofluorescence Microscopy

Immunofluorescence experiments were performed as described previously [50] using the following antibody dilutions: anti-NSP5 guinea pig serum 1∶1,000; anti-NSP4 mouse monoclonal antibody 1∶500 (clone B4-2, gently provided by H. B. Greenberg, School of Medicine, Stanford University, Palo Alto, CA, USA); anti-VP4 guinea pig serum 1∶200 (produced as described previously [51]); anti-VP6 mouse monoclonal antibody 1∶1,000 (clone 4B2D2, gently provided by J. L. Zambrano and F. Liprandi, Instituto Venezolano de Investigaciones Científicas, Caracas, Venezuela); anti-LC3B rabbit antibody (1∶200, Sigma); anti-SV5 mouse monoclonal antibody, 1∶500; Alexa Fluor 488-conjugated anti-mouse (1∶500, Life Technologies), Alexa Fluor 647-conjugated anti-guinea pig (1∶1,000, Life Technologies), Alexa Fluor 546-conjugated anti-rabbit (1∶500, Life Technologies) secondary antibodies. To avoid cross-reactivity of the anti-rabbit antibody with the anti-NSP5 serum, antibody reactions of the experiment shown in Figure 5B were performed sequentially, using first the anti-LC3 and the relative secondary antibody and afterwards the anti-NSP5 and the relative secondary antibody. Cell nuclei were stained with 2 µg/ml Hoechst 33342 (Molecular Probes, Life Technologies). Samples were analyzed by confocal microscopy (Zeiss LSM510 equipped with a 100× NA 1.3 objective or Zeiss LSM510 Meta equipped with a 63× NA 1.4 objective).

For the quantification of viroplasms, images were acquired using an ImageXpress Micro automated high-content screening microscope (Molecular Devices) equipped with a 20× objective; a total of 9 fields were acquired, corresponding to about 1,000 cells analyzed per experimental condition and replicate. Automated image analysis of viroplasm formation was performed by MetaXpress software (Molecular Devices) using the Transfluor application module, which identifies cell nuclei (blue channel) and quantifies the number of fluorescent spots in each cell (green channel).

### Transmission Electron Microscopy

MA104 cells were seeded at 8×10^4^ cells in a 2 cm^2^ well onto sapphire discs and infected with simian rotavirus SA11 (MOI, 250 VFU/cell, according to virus titration described in [40]). Cells were fixed with 2.5% glutaraldehyde in 100 mM Na/K-phosphate buffer, pH 7.4 for 1 h at 4°C and kept into 100 mM Na/K-phosphate buffer overnight at 4°C. Afterwards, samples were post-fixed with 1% osmium tetroxide in 100 mM Na/K-phosphate buffer for 1 h at 4°C, dehydrated in a graded ethanol series starting at 70% followed by two changes in acetone and embedded in epon. Ultrathin sections (60–80 nm) were cut and stained with uranyl-acetate and lead citrate before analysis in a transmission electron microscope (CM12, Philips) equipped with a CCD camera (Ultrascan 1000, Gatan) at an acceleration of 100 kV. For the quantification of autophagosomes, pictures from perinuclear and from cell periphery area were acquired for 25 cells in each experimental point by transmitted electron microscopy. The mean of autophagosomes were quantified per cytosolic area through a multipurpose test system [52] with the following formula: 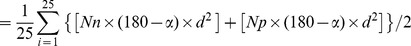



N_n_ correspond to the number of autophagosomes in the perinuclear area, N_p_ correspond to number of autophagosomes in the cell periphery; α correspond to the number of test lines outside cytosolic region and d is the test line length (µm). The experiment was repeated three times, the mean significances were analyzed by two-tailed paired t-test and plotted using Graph Pad Prism (Graph Pad Software, Inc.).

## Supporting Information

Figure S1 **SV5-LC3 validation as a marker of autophagosomes.** A) Confocal immunofluorescence of CQ-treated MA104 cells transiently over-expressing the pSV5-LC3 construct. Autophagosomes were visualized with an anti-LC3 antibody (red) and with an anti-SV5 antibody (green). CQ was used to increase the number of autophagosomes. Single optical sections are shown. Scale bar is 5 µm. Images are representative of three independent experiments in which at least 150 cells per each experimental condition were analyzed. B) Western blot of extracts from MA104 cells upon different treatments: infection with OSU (13 hpi), incubation with CQ (50 µM), or starvation.
(TIF)

Figure S2 **Impairment of LC3 lipidation upon depletion of Atg7.** Western blot of extracts from non-infected MA104 cells transfected with the indicated siRNAs. At 48 h after transfection, cells were treated or not with RAP (0.1 µM) for 12 h. NT: control non-targeting siRNA.
(TIF)
