# Supplementary figures and images for: Rotavirus Increases Levels of Lipidated LC3 Supporting Accumulation of Infectious Progeny Virus without Inducing Autophagosome Formation
Source: PLoS One. 2014 Apr 15;9(4):e95197. doi: 10.1371/journal.pone.0095197 (PMC3988245; doi:10.1371/journal.pone.0095197)

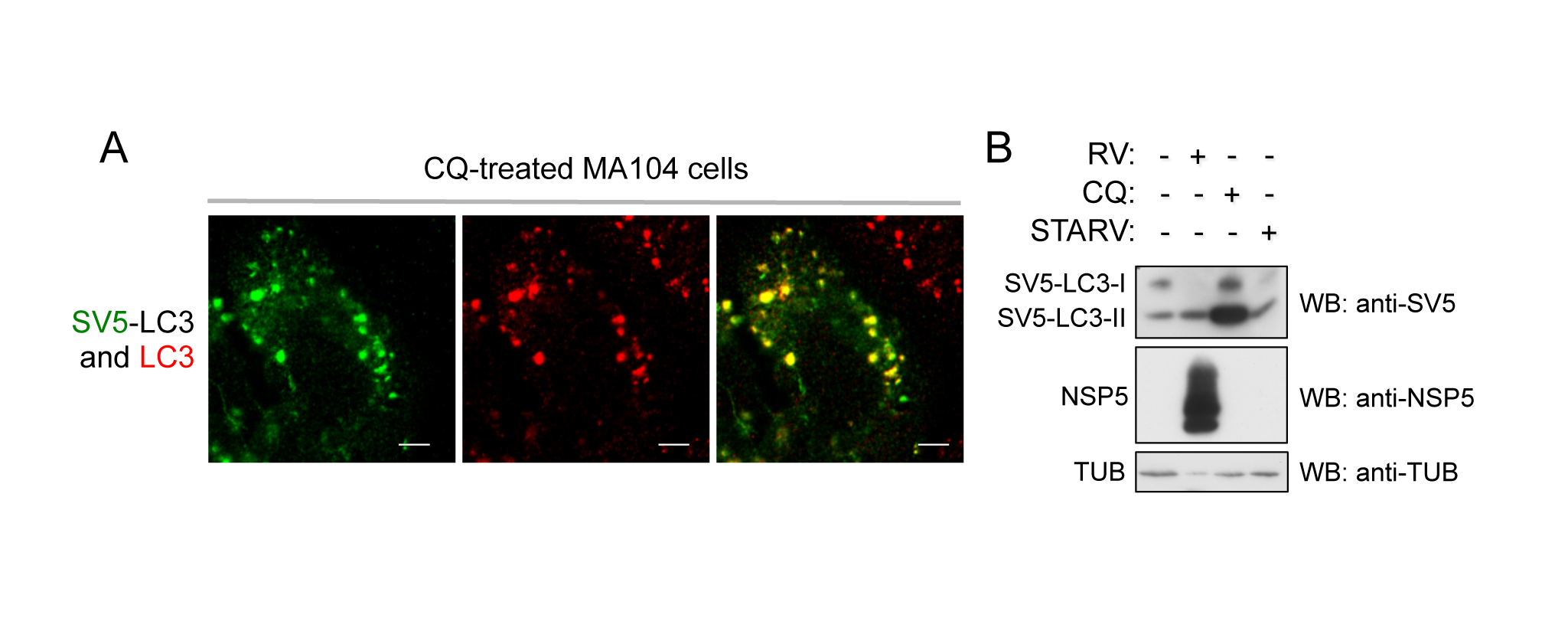

Supplement: Figure S1 — SV5-LC3 validation as a marker of autophagosomes. A) Confocal immunofluorescence of CQ-treated MA104 cells transiently over-expressing the pSV5-LC3 construct. Autophagosomes were visualized with an anti-LC3 antibody (red) and with an anti-SV5 antibody (green). CQ was used to increase the number of autophagosomes. Single optical sections are shown. Scale bar is 5 µm. Images are representative of three independent experiments in which at least 150 cells per each experimental condition were analyzed. B) Western blot of extracts from MA104 cells upon different treatments: infection with OSU (13 hpi), incubation with CQ (50 µM), or starvation. (TIF) [file pone.0095197.s001.tif]

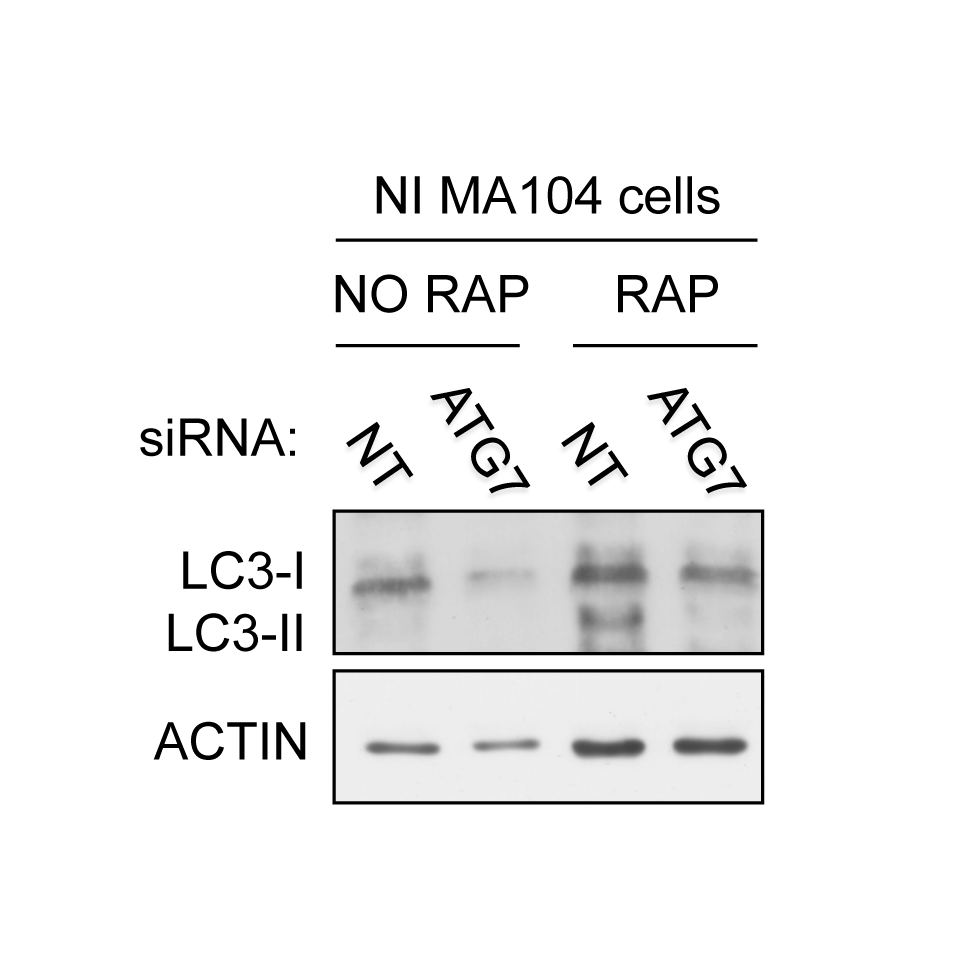

Supplement: Figure S2 — Impairment of LC3 lipidation upon depletion of Atg7. Western blot of extracts from non-infected MA104 cells transfected with the indicated siRNAs. At 48 h after transfection, cells were treated or not with RAP (0.1 µM) for 12 h. NT: control non-targeting siRNA. (TIF) [file pone.0095197.s002.tif]
